# The mycotoxin phomoxanthone A disturbs the form and function of the inner mitochondrial membrane

**DOI:** 10.1038/s41419-018-0312-8

**Published:** 2018-02-19

**Authors:** Philip Böhler, Fabian Stuhldreier, Ruchika Anand, Arun Kumar Kondadi, David Schlütermann, Niklas Berleth, Jana Deitersen, Nora Wallot-Hieke, Wenxian Wu, Marian Frank, Hendrik Niemann, Elisabeth Wesbuer, Andreas Barbian, Tomas Luyten, Jan B. Parys, Stefanie Weidtkamp-Peters, Andrea Borchardt, Andreas S. Reichert, Aida Peña-Blanco, Ana J. García-Sáez, Samuel Itskanov, Alexander M. van der Bliek, Peter Proksch, Sebastian Wesselborg, Björn Stork

**Affiliations:** 1 Institute of Molecular Medicine I, Medical Faculty, Heinrich Heine University Düsseldorf, 40225 Düsseldorf, Germany; 2 Institute of Biochemistry and Molecular Biology I, Medical Faculty, Heinrich Heine University Düsseldorf, 40225 Düsseldorf, Germany; 3 Institute of Pharmaceutical Biology and Biotechnology, Faculty of Mathematics and Natural Sciences, Heinrich Heine University Düsseldorf, 40225 Düsseldorf, Germany; 4 Institute of Anatomy I, Medical Faculty, Heinrich Heine University Düsseldorf, 40225 Düsseldorf, Germany; 5 Laboratory of Molecular and Cellular Signaling, Department of Cellular and Molecular Medicine, KU Leuven, 3000 Leuven, Belgium; 6 Center for Advanced Imaging, Faculty of Mathematics and Natural Sciences, Heinrich Heine University Düsseldorf, 40225 Düsseldorf, Germany; 7 Interfaculty Institute of Biochemistry, Eberhard Karls University Tübingen, 72076 Tübingen, Germany; 8 Department of Biological Chemistry, David Geffen School of Medicine at UCLA, Los Angeles, CA 90095 USA

## Abstract

Mitochondria are cellular organelles with crucial functions in the generation and distribution of ATP, the buffering of cytosolic Ca^2+^ and the initiation of apoptosis. Compounds that interfere with these functions are termed mitochondrial toxins, many of which are derived from microbes, such as antimycin A, oligomycin A, and ionomycin. Here, we identify the mycotoxin phomoxanthone A (PXA), derived from the endophytic fungus *Phomopsis longicolla*, as a mitochondrial toxin. We show that PXA elicits a strong release of Ca^2+^ from the mitochondria but not from the ER. In addition, PXA depolarises the mitochondria similarly to protonophoric uncouplers such as CCCP, yet unlike these, it does not increase but rather inhibits cellular respiration and electron transport chain activity. The respiration-dependent mitochondrial network structure rapidly collapses into fragments upon PXA treatment. Surprisingly, this fragmentation is independent from the canonical mitochondrial fission and fusion mediators DRP1 and OPA1, and exclusively affects the inner mitochondrial membrane, leading to cristae disruption, release of pro-apoptotic proteins, and apoptosis. Taken together, our results suggest that PXA is a mitochondrial toxin with a novel mode of action that might prove a useful tool for the study of mitochondrial ion homoeostasis and membrane dynamics.

## Introduction

Mitochondria are cellular organelles that are crucial to almost all eukaryotic organisms. Among their most important functions are generation and distribution of ATP, buffering of cytosolic Ca^2+^ and, in animal cells, initiation of apoptosis. Disturbance of these or other functions by mitochondrial toxins can lead to cellular stress and cell death^1,2^.

Mitochondria produce ATP through oxidative phosphorylation (OXPHOS), which depends on the electron transport chain (ETC) embedded in the inner mitochondrial membrane (IMM). The ETC pumps protons out of the mitochondrial matrix and into the mitochondrial intermembrane space. This generates a proton gradient (ΔpH_m_) and, consequently, a membrane potential (ΔΨ_m_) across the IMM. The ΔΨ_m_ is then used to drive the mitochondrial ATP synthase^3^.

To provide all regions within the cell with sufficient ATP, mitochondria often form a network that constantly undergoes balanced fission and fusion. This allows remodelling of the network as well as removal and recycling of damaged mitochondria through mitophagy^1,4,5^. Excessive fission can be triggered by mitochondrial toxins that cause loss of ΔΨ_m_, such as the protonophore carbonyl cyanide *m*-chlorophenyl hydrazone (CCCP)^6^.

The ΔΨ_m_ also plays a role in the mitochondrial buffering of cytosolic Ca^2+^. Normally, the cytosol of a typical animal cell contains only a very low Ca^2+^ concentration ([Ca^2+^]_cyt_, ~0.1 µM), whereas the concentration of Ca^2+^ within the endoplasmic reticulum ([Ca^2+^]_ER_, > 100 µM) or outside the cell ([Ca^2+^]_ext_, > 1000 µM) is up to 10,000-fold higher^2^. In response to certain stimuli, Ca^2+^ channels in the ER and/or the plasma membrane open to release Ca^2+^ into the cytosol as a second messenger. Mitochondria contribute to removal of cytosolic Ca^2+^ by uptake into their matrix via ΔΨ_m_-driven Ca^2+^ transporters. After that, a slow, regulated efflux moves the Ca^2+^ out of the matrix and into the cristae, which are folds in the IMM, from where it is slowly released and shuttled back to the ER^2,7–9^. A separate mechanism through which Ca^2+^ can cross the IMM is the mitochondrial permeability transition pore (mPTP), which can open irreversibly in response to severe mitochondrial stress. The mPTP directly connects the mitochondrial matrix with the cytosol to allow the free exchange of molecules up to 1.5 kDa in size, including Ca^2+^. Irreversible mPTP opening leads to release of mitochondrial Ca^2+^, loss of ΔΨ_m_, swelling of the matrix and eventually mitochondrial outer membrane permeabilisation (MOMP)^10,11^.

In animal cells, MOMP initiates apoptosis. Several proteins normally contained in the cristae attain a pro-apoptotic function if they pass the outer mitochondrial membrane (OMM) and are released into the cytosol. Among these proteins are cytochrome c (CYCS), SMAC (DIABLO) and OMI (HTRA2). Cytosolic CYCS becomes part of the caspase-activating apoptosome complex, while DIABLO and HTRA2 bind and inhibit the inhibitor of apoptosis proteins (IAPs), thus attenuating their inhibition of caspases^1^. MOMP can be caused either passively through rupture of the OMM, such as triggered by the mPTP, or actively through the formation of pores in the OMM by the pro-apoptotic proteins BAK and BAX, which can be induced in response to severe cellular stress^12^.

A variety of mitochondrial toxins with different effects and molecular targets is known today^13^. Several of these toxins are natural products, such as the *Streptomyces*-derived ETC inhibitor antimycin A and the ATP synthase inhibitor oligomycin A.

Phomoxanthone A and B (PXA and PXB) are natural products named after the fungus *Phomopsis*, from which they were first isolated, and after their xanthonoid structure (Fig. S1). PXA is a homodimer of two acetylated tetrahydroxanthones symmetrically linked at C-4,4’, whereas PXB is structurally almost identical but asymmetrically linked at C-2,4’. Both possess antibiotic activity against diverse organisms from all biological kingdoms. Originally described in 2001, PXA and PXB were tested against the protozoan *Plasmodium falciparum*, the Gram-positive *Mycobacterium tuberculosis*, and three animal cell lines. In all of these organisms, both PXA and PXB showed significant cytotoxic activity, with PXA being more toxic in every case^14^. A later study in different organisms produced similar results, showing that PXA inhibits the growth of the Gram-positive *Bacillus megaterium*, the alga *Chlorella fusca*, and the fungus *Ustilago violacea*^15^.

We previously showed that PXA induces apoptosis in human cancer cell lines. Signs of apoptosis were observed as early as after 4 h of treatment with low micromolar doses of PXA^16,17^. However, the mechanism by which PXA causes apoptosis or cytotoxicity in general has never before been investigated.

The aim of this study was to elucidate the mechanism through which PXA exerts its toxicity. Following our initial results, we hypothesised that PXA directly affects the mitochondria and thus investigated its effects on the ETC, ΔΨ_m_, ATP production, Ca^2+^ buffering, and mitochondrial morphology. It appears that PXA is a mitochondrial toxin that specifically affects the IMM, leading to loss of ΔΨ_m_, ETC inhibition, Ca^2+^ efflux, mitochondrial fragmentation, cristae disruption, and finally to the release of mitochondrial pro-apoptotic factors.

## Results

### PXA induces Ca^2+^ release from an intracellular store

**Fig. 1:**
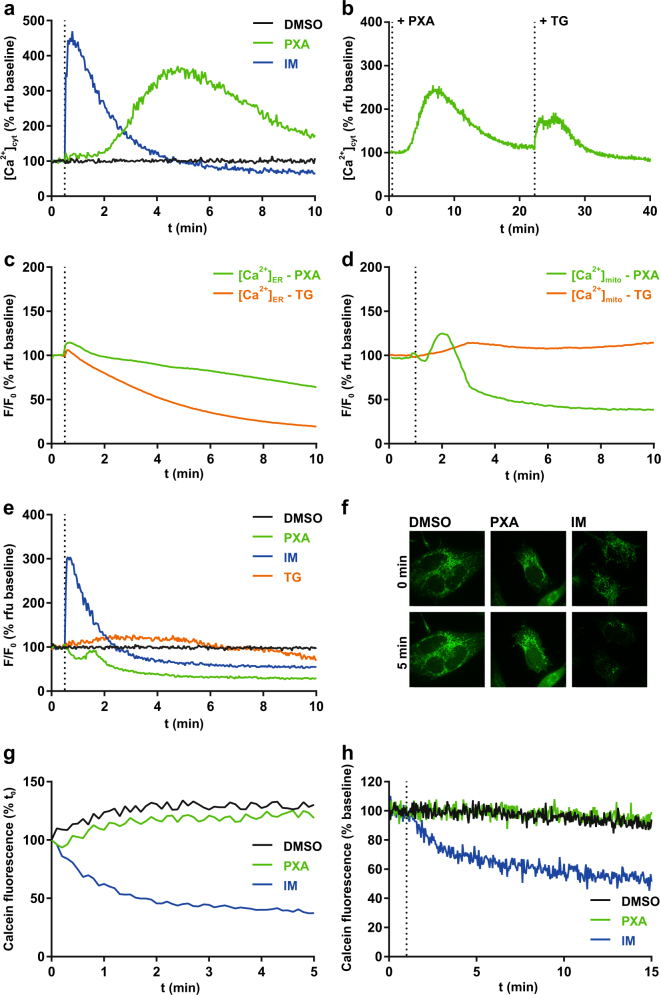
PXA causes an increase of [Ca^2+^]_cyt_ and a release of [Ca^2+^]_mito_ but not [Ca^2+^]_ER_. **a** Live measurement of the effect of PXA (10 µM) on [Ca^2+^]_cyt_ in Ramos cells, where DMSO (0.1% v/v) was used as vehicle control and ionomycin (IM; 2 µM) was used as positive control, and **b** live measurement of [Ca^2+^]_cyt_ after PXA followed by thapsigargin (TG; 10 µM). Measurements were performed by flow cytometry using the Ca^2+^-sensitive fluorescent probe Fluo-4-AM (Ex 488 nm, Em 530 ± 30 nm) in the absence of extracellular Ca^2+^ by maintaining the cells in Krebs-Ringer buffer containing 0.5 mM EGTA during measurement. **c**,** d** Comparison of the effect of PXA (10 µM) and thapsigargin (TG; 1 µM) on either [Ca^2+^]_ER_ or [Ca^2+^]_mito_ as measured by the Ca^2+^-sensitive fluorescent protein CEPIA targeted to the respective organelle in HeLa cells. All traces were normalised (*F*/*F*_0_) where *F*_0_ is the starting fluorescence of each trace. **e** Comparison of the effect of PXA (10 µM), ionomycin (IM; 2 µM), and thapsigargin (TG; 1 µM) on [Ca^2+^]_mito_ in Ramos cells stably transfected with the Ca^2+^-sensitive ratiometric fluorescent protein mito-Pericam. DMSO (0.1% v/v) was used as vehicle control. *F*/*F*_0_ is the ratio of fluorescence with excitation at 488 nm (high [Ca^2+^]) to 405 nm (low Ca^2+^]). **f**, **g** Live imaging and quantification of the effect of PXA (10 µM) on mPTP opening in HeLa cells as measured by mitochondrial calcein fluorescence using the calcein/cobalt quenching method. DMSO (0.1% v/v) was used as vehicle control and ionomycin (IM; 2 µM) was used as positive control. Mitochondrial calcein fluorescence was quantified. **h** Additional live measurement of the effect of PXA on mPTP opening in Ramos cells by the calcein/cobalt quenching method using flow cytometry

To determine how PXA induces apoptosis, we analysed its effect on cellular Ca^2+^ levels since ionic imbalance can be an apoptotic trigger. Treatment of Ramos cells with PXA resulted in a strong, steady increase of [Ca^2+^]_cyt_ (Fig. 1a). Interestingly, there was a delay of about 2–5 min between addition of PXA and increase in [Ca^2+^]_cyt_. Since this pattern of Ca^2+^ release is similar to that caused by the tyrosine phosphatase inhibitor pervanadate (VO_4_^3−^) (Fig. S2a), and since tyrosine phosphatase inhibition can induce apoptosis, we tested the effect of PXA on tyrosine phosphorylation. However, in contrast to pervanadate, we could not detect any effect (Fig. S2b). In a broader picture, PXA had no inhibitory effect on any of 141 protein kinases against which we tested it (Table S1).

We next tried to determine the origin of the released Ca^2+^. Since PXA increases [Ca^2+^]_cyt_ even in the absence of extracellular Ca^2+^, we tested if it releases Ca^2+^ from the ER. Using thapsigargin, which causes a net efflux of Ca^2+^ from the ER, we could induce an increase in [Ca^2+^]_cyt_ even after PXA-inducible Ca^2+^ stores were depleted (Fig. 1b), suggesting that the Ca^2+^ released by PXA at least partially originates from a source other than the ER.

### PXA induces Ca^2+^ release mainly from the mitochondria

To quantify the effect of PXA on Ca^2+^ stores, we used HeLa cells expressing CEPIA Ca^2+^ probes targeted to either the ER or the mitochondria. Although PXA provoked some Ca^2+^ release from the ER, it was much slower and weaker than that evoked by thapsigargin (Fig. 1c). Mitochondria, however, were quickly and severely depleted (Fig. 1d). This effect of PXA on mitochondrial Ca^2+^ was confirmed in Ramos cells, making use of the Ca^2+^ probe Pericam (Fig. 1e).

### Mitochondrial Ca^2+^ release caused by PXA is independent from the mPTP

**Fig. 2:**
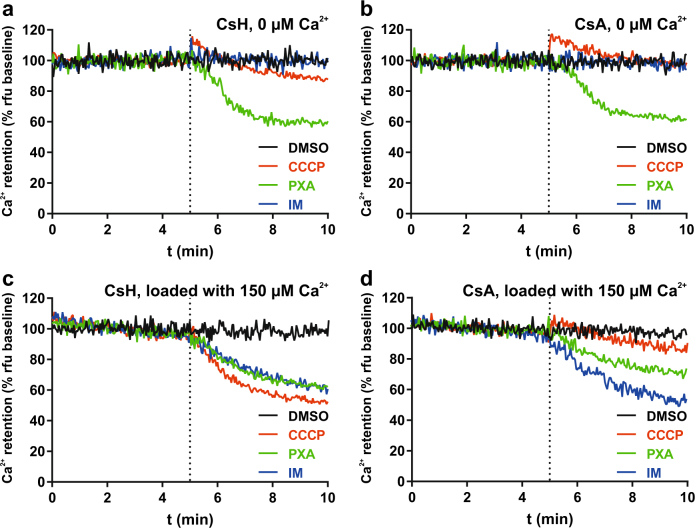
Live measurement of the effect of PXA on mitochondrial Ca^2+^ retention capacity. Isolated mitochondria were stained with calcium green AM and maintained in the presence of either (**a**,** c**) CsH or (**b**,** d**) CsA, and fluorescence was measured either (**a**, **b**) directly after staining or (**c**, **d**) after first loading the mitochondria with 150 µM Ca^2+^

Large-scale Ca^2+^ efflux from the mitochondria can result from persistent opening of the mPTP. We thus tested whether PXA induces mPTP opening by using the cobalt/calcein method, comparing PXA to the mPTP inducer ionomycin (IM). While IM caused a strong decrease in mitochondrial calcein fluorescence as expected, PXA had no observable effect (Fig. 1f, g; Supplementary Movies S1–S3). Similar results were obtained by further live measurement using flow cytometry (Fig. 1h). In addition, we tested whether the mPTP inhibitor cyclosporin A (CsA) can prevent the mitochondrial Ca^2+^ release caused by PXA. We measured mitochondrial Ca^2+^ retention capacity in isolated mitochondria, comparing PXA to IM and CCCP. This was done in either normal isolated mitochondria or mitochondria loaded with Ca^2+^, and in the presence of either CsA or its derivative cyclosporin H (CsH), which does not affect the mPTP (Fig. 2)^18^. While PXA caused a decrease in calcium green fluorescence, indicating Ca^2+^ release, under every condition, i.e., regardless of Ca^2+^ loading and also in the presence of CsA, IM had an observable effect only in loaded mitochondria, but also regardless of CsA. On the other hand, CCCP caused a release of Ca^2+^ only in the presence of CsH but not CsA, indicating that CCCP-induced Ca^2+^ release does indeed depend on the mPTP, unlike that induced by PXA. Taken together, these results indicate that PXA causes mitochondrial Ca^2+^ release largely independent from the mPTP.

### PXA depolarises the mitochondria but does not uncouple cellular respiration

**Fig. 3:**
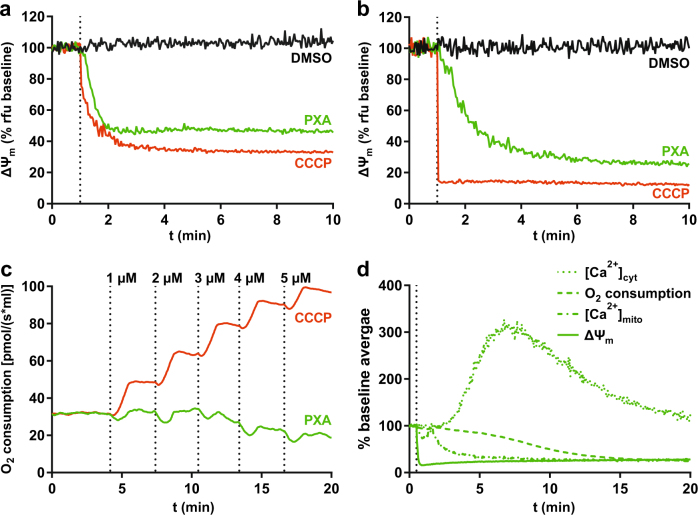
PXA dissipates the mitochondrial membrane potential (ΔΨ_m_) but does not increase cellular respiration. **a** Live measurement of the effect of PXA (10 µM) on ΔΨ_m_ in Ramos cells. Measurements were performed by flow cytometry using the ΔΨ_m_-sensitive fluorescent probe TMRE (Ex 488 nm, Em 575 ± 26 nm). A decrease in TMRE fluorescence corresponds to a decrease in ΔΨ_m_. The protonophore carbonyl cyanide *m*-chlorophenyl hydrazone (CCCP; 10 µM) was used as positive control for mitochondrial depolarisation. **b** Live measurement of the effect of PXA (10 µM) ΔΨ_m_ in isolated mitochondria. Measurements were performed by flow cytometry using the ΔΨ_m_-sensitive fluorescent probe TMRM (Ex 488 nm, Em 575 ± 26 nm). The mitochondria were maintained in the presence of 1.6 µM CsH to prevent passive TMRM leakage. **c** Titration of PXA to determine the effect on cellular respiration in Ramos cells as measured by oxygen consumption. CCCP was used as positive control for increase of respiration. Measurement of extracellular [O_2_] was performed using an oxygraph. **d** Comparison of the kinetics of the effects of PXA on [Ca^2+^]_cyt_ (as measured by Fluo-4-AM fluorescence), [Ca^2+^]_mito_ (as measured by mito-Pericam fluorescence), O_2_ consumption (as measured by changes in extracellular [O_2_]) and ΔΨ_m_ (as measured by TMRE fluorescence). Graphs were partially adapted from other figures

A change in [Ca^2+^]_mito_ likely correlates with changes in other mitochondrial ion gradients. Uptake of Ca^2+^ into the mitochondrial matrix is driven by ΔΨ_m_. We thus analysed the effect of PXA on ΔΨ_m_. Indeed, PXA caused immediate mitochondrial depolarisation similar to CCCP, both in whole cells and isolated mitochondria (Fig. 3a, b). The EC_50_ for PXA-induced loss of ΔΨ_m_ in Ramos cells was determined to be 1.1 ± 0.3 µM (Fig. S3). The key contributor to ΔΨ_m_ is ΔpH_m_, which is maintained via cellular respiration by consumption of O_2_. If the PXA-induced loss of ΔΨ_m_ was caused by loss of ΔpH_m_ downstream of the ETC, as in case of CCCP, it would be accompanied by an increase in respiration to compensate for the loss. Therefore, we measured cellular O_2_ consumption upon increasing concentrations of either PXA or CCCP. As expected, CCCP caused a dose-dependent increase in O_2_ consumption. However, in contrast to CCCP, PXA caused no increase but rather a slight decrease in O_2_ consumption (Fig. 3c). An overview of the kinetics of the effects of PXA on [Ca^2+^]_cyt_, [Ca^2+^]_mito_, O_2_ consumption and ΔΨ_m_ is presented in Fig. 3d.

### PXA inhibits cellular respiration by disrupting the electron transport chain

**Fig. 4:**
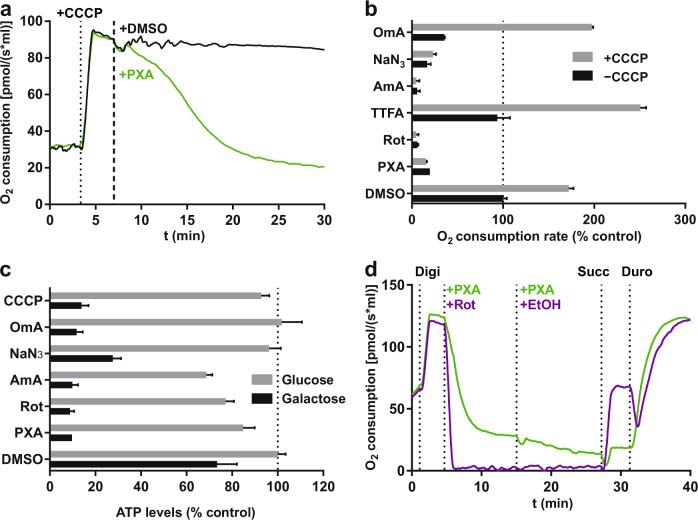
Effect of PXA on electron transport chain and OXPHOS. **a** Live measurement of the effect of PXA on uncoupled cellular respiration in intact Ramos cells. Cells were first treated with 1 µM CCCP to uncouple respiration and then with either 10 µM PXA or 0.1% v/v DMSO (vehicle control) to test for inhibition. Measurement of extracellular [O_2_] was performed using an oxygraph. **b** Comparative measurements of the effect of PXA and other compounds on basal and uncoupled respiration in Ramos cells. Respiration was uncoupled by CCCP (1 µM). PXA (10 µM) was compared to the complex-specific ETC inhibitors rotenone (Rot; complex I; 10 µM), thenoyltrifluoroacetone (TTFA; complex II; 10 µM), antimycin A (AmA; complex III; 10 µM), azide (NaN_3_; complex IV; 1 mM) and oligomycin A (OmA; complex V; 10 µM). Measurement was performed in a microplate reader using the fluorescence-based *MITO-ID® Extracellular O2 Sensor Kit (High Sensitivity)* (Enzo). **c** Comparative measurements of the effect of PXA and other compounds on ATP levels in Ramos cells after 120 min of treatment. PXA (10 µM) was compared to complex-specific ETC inhibitors (see above) as well as CCCP (1 µM). Measurement was performed in a microplate reader using the luminescence-based *Mitochondrial ToxGlo™ Assay* (Promega). Cells were incubated in full growth medium containing either glucose or galactose as the only available sugar. Galactose alone forces the cells to resort exclusively to OXPHOS for ATP synthesis. **d** Live measurement of mitochondrial respiration in Ramos cells permeabilized by digitonin (Digi; 5 µg/ml). The effect of PXA (first 1 µM, then increased to 10 µM as indicated) was compared to that of the known complex I inhibitor rotenone (Rot; 1 µM). To specifically induce complex II and III of the electron transport chain, succinate (Succ; 10 mM) and duroquinol (Duro; 1 mM) were used, respectively. Measurement of extracellular [O_2_] was performed using an oxygraph

Since PXA had a moderate inhibitory effect on cellular O_2_ consumption under basal conditions, we next measured O_2_ consumption after the respiration rate was first increased by CCCP. Here, treatment with PXA caused a strong decrease in O_2_ consumption to levels below baseline (Fig. 4a). It thus appeared likely that PXA, unlike CCCP, is not an inducer but rather an inhibitor of cellular respiration and of the ETC. We, therefore, compared PXA to known ETC inhibitors: rotenone (complex I), thenoyltrifluoroacetone (TTFA; complex II), antimycin A (complex III), sodium azide (NaN_3_; complex IV) and oligomycin A (complex V / ATP synthase). In an O_2_ consumption assay, PXA caused a strong decrease in cellular respiration, both after CCCP treatment and under basal conditions, similar to that caused by rotenone, antimycin A and azide (Fig. 4b). Oligomycin A expectedly inhibited respiration under basal conditions but not after CCCP treatment since CCCP uncouples respiration from ATP synthesis. TTFA did not have a significant effect, probably because complex II is not involved in respiration if complex I substrates are available^19^.

Since a functional ETC is required for ATP synthesis by OXPHOS, we also compared PXA to known ETC inhibitors in this context. Indeed, PXA as well as all tested ETC inhibitors strongly reduced cellular ATP levels if galactose was the only available sugar and ATP had to be synthesised via OXPHOS instead of glycolysis (Fig. 4c). Thus assuming that PXA targets the ETC, we tried to determine if it specifically inhibits one of the ETC complexes. This experiment was performed in permeabilized cells, comparing PXA to rotenone. Succinate, which induces complex II-dependent respiration only if complex I is inhibited, alleviated rotenone-induced inhibition of O_2_ consumption but had only a marginal effect in PXA-treated cells. In contrast, duroquinol, which induces complex III-dependent respiration, increased O_2_ consumption in both PXA-treated as well as rotenone-treated cells back to levels before inhibition (Fig. 4d). These data suggest that PXA might either affect both complex I and II or the shuttling of electrons between complex I/II and III.

### Comparison of PXA with other ETC inhibitors

**Fig. 5:**
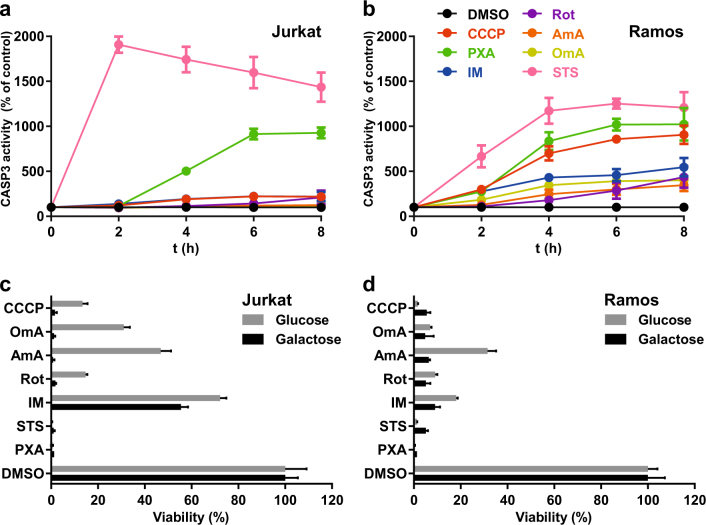
Comparative measurements of the effect of PXA and other compounds on apoptosis induction and cytotoxicity in Jurkat and Ramos cells. PXA (10 µM) was compared to the protonophore carbonyl cyanide *m*-chlorophenyl hydrazone (CCCP; 10 µM), the Ca^2+^ ionophore ionomycin (IM; 10 µM), the apoptosis inducer staurosporine (STS; 10 µM), and the complex-specific ETC inhibitors rotenone (Rot; complex I; 10 µM), antimycin A (AmA; complex III; 10 µM) and oligomycin A (OmA; complex V; 10 µM). Data shown are the means of three independent experiments; error bars = SD. **a**,** b** Measurement of apoptosis induction based on cleavage of the pro-fluorescent CASP3 substrate Ac-DEVD-AMC, which results in release of AMC (Ex 360 nm, Em 450 nm), as an indicator of apoptosis. **c**, **d** Measurement of cytotoxicity after 24 h of treatment in the presence of either glucose or galactose as the only available sugar. Galactose alone forces the cells to resort exclusively to OXPHOS for ATP synthesis. Measurement was performed in a microplate reader using the MTT viability assay

While PXA inhibits the ETC as well as ATP synthesis, it differs from the other ETC inhibitors used in this study concerning its effects on Ca^2+^ and ΔΨ_m_. Unlike PXA, neither CCCP nor any of the tested ETC inhibitors with the exception of antimycin A caused a noticeable release of Ca^2+^ (Fig. S4a), and that caused by antimycin A was much weaker and had an earlier but slower onset than the one caused by PXA. Similarly, while both CCCP and PXA induced a strong and immediate decrease in ΔΨ_m_, none of the other ETC inhibitors except antimycin A had any effect on ΔΨ_m_, and that of antimycin A was much slower and weaker (Fig. S4b). Since we previously showed that PXA is cytotoxic and induces apoptosis, we also compared it to CCCP, ETC inhibitors, IM (control for Ca^2+^ release) and staurosporine (control for cytotoxicity/apoptosis) in these regards. PXA, staurosporine and CCCP strongly induced apoptosis, while the ETC inhibitors and IM were much weaker inducers in Ramos cells and did not noticeably induce apoptosis at all in Jurkat cells (Fig. 5a, b). Dependency on OXPHOS for ATP synthesis, which considerably increased the toxicity of the ETC inhibitors, appeared to have no effect on the toxicity of PXA or staurosporine (Fig. 5c, d). These observations indicate that PXA probably causes cytotoxicity in general and apoptosis in particular not via its effects on the ETC, ΔΨ_m_, or [Ca^2+^]_mito_, but rather that all of these events might have a common cause further upstream.

### PXA causes irreversible cleavage of OPA1 mediated by OMA1 but not YME1L1

**Fig. 6:**
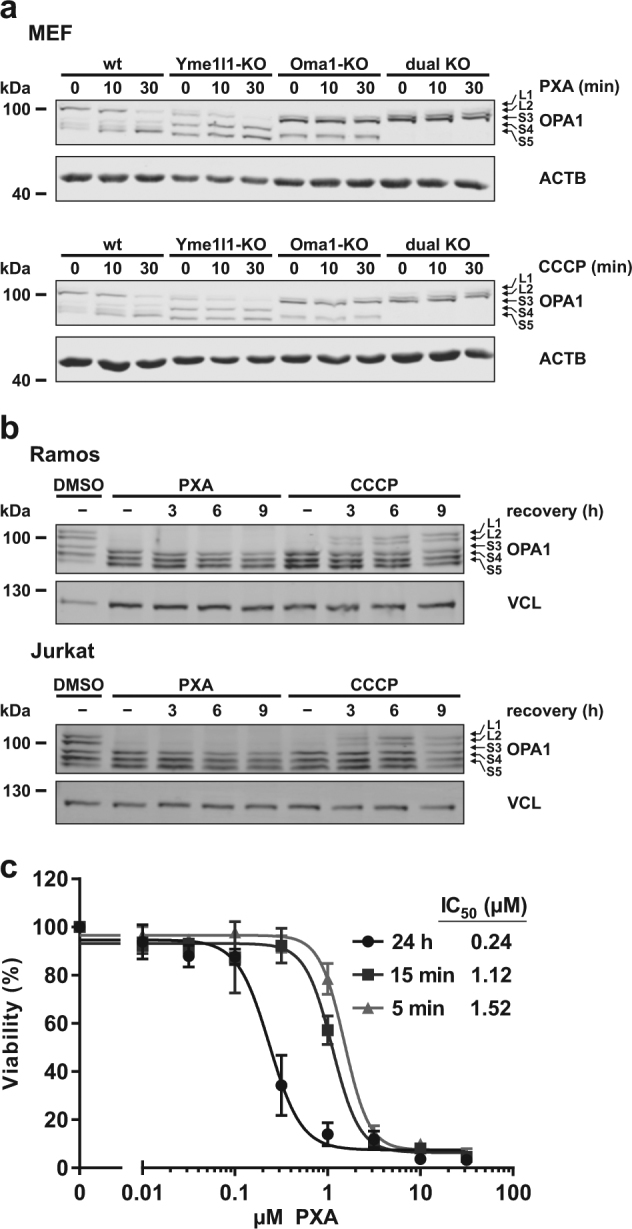
PXA causes irreversible OMA1-dependent OPA1 processing and primes cells for death. **a** Effect of PXA (10  µM) and of the protonophore carbonyl cyanide *m*-chlorophenyl hydrazone (CCCP; 10  µM) on OPA1 processing in cells deficient for the OPA1 protease OMA1 and/or YME1L1. Proteins were detected by immunoblot. Beta-actin (ACTB) was used as loading control. **b** Examination of the recovery of long OPA1 forms in Ramos or Jurkat cells after 5 min of treatment with either PXA (10 µM) or CCCP (10 µM) followed by removal of the substance and a recovery period of up to 9 h. Proteins were detected by immunoblot. Vinculin (VCL) was used as loading control. **c** Comparison of the effect of PXA on cell viability if the stimulus was removed after treatment. Measurement was performed in a microplate reader using the MTT viability assay. Data shown are the means of three independent experiments; error bars = SD

Several stress conditions including loss of ΔΨ_m_ and low levels of ATP can induce cleavage of the IMM fusion regulator OPA1 by the protease OMA1 (ref. ^20^). Additionally, OPA1 is also cleaved by the protease YME1L1, resulting in fragments of different size. We treated MEF cells deficient for either one or both of these proteases with either PXA or CCCP. We observed that PXA, like CCCP, caused stress-induced OPA1 cleavage that was dependent on OMA1, whereas expression of YME1L1 did not have any visible effect on PXA-induced OPA1 cleavage (Fig. 6a). Similarly to their effect in MEF cells, PXA and CCCP-induced cleavage of OPA1 in Ramos and Jurkat cells within minutes. Interestingly, and unlike CCCP, removal of PXA did not enable recovery of the long OPA1 forms (Fig. 6b). This prompted us to also investigate the effects of removal of PXA on cytotoxicity. Indeed, though PXA was about fivefold less toxic if removed after a few minutes, it still irreversibly primed the cells for death (Fig. 6c). It, thus, appears that at least some of the effects of PXA on the cells are irreversible.

### PXA induces fragmentation of the inner but not of the outer mitochondrial membrane independently of OMA1, OPA1 and DRP1

**Fig. 7:**
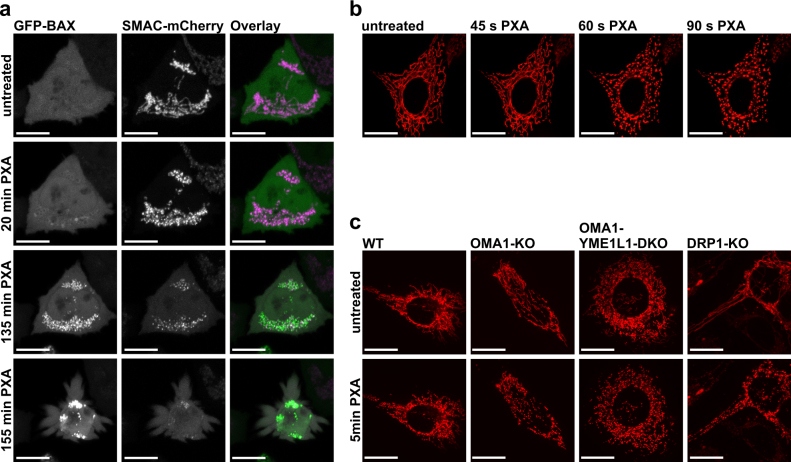
Effects of PXA on mitochondrial integrity and morphology. **a** Confocal live imaging of a HCT116 cell expressing GFP-BAX (green) and SMAC-mCherry (magenta) after treatment with 10 µM PXA. **b** Confocal live imaging of a HeLa cell stably expressing the fluorescent dye mito-DsRed, which localises to the mitochondrial matrix, after treatment with 10 µM PXA. **c** Confocal live imaging of MEF cells (WT, OMA1-KO, OMA1-YME1L1-DKO and DRP1-KO) before and after treatment with 10 µM PXA. The cells are stably expressing the fluorescent dye mito-DsRed, which localises to the mitochondrial matrix. All scale bars are equivalent to 10 µm

Excessive processing of OPA1 by OMA1 changes mitochondrial cristae morphology, resulting in the release of pro-apoptotic factors such as CYCS and SMAC. We, therefore, investigated the effects of PXA on SMAC localisation and on recruitment of BAX to the OMM. We observed that PXA indeed induced recruitment of GFP-BAX to the mitochondria, with concurrent release of SMAC-mCherry into the cytosol, within about 2–3 h (Fig. 7a and Supplementary Movie S4; quantification shown in Fig. S5). OPA1 processing by OMA1 also prevents IMM fusion and, if excessive, results in mitochondrial fragmentation. Intriguingly, PXA caused rapid fragmentation of the mitochondrial network within minutes (Fig. 7b), and independent of the cells’ OMA1 or YME1L1 status (Fig. 7c, left panels; Supplementary Movies S5–S7). The persistence of PXA-induced mitochondrial fragmentation in OMA1-YME1L1 DKO cells indicates that this process is independent of OPA1 cleavage.

**Fig. 8:**
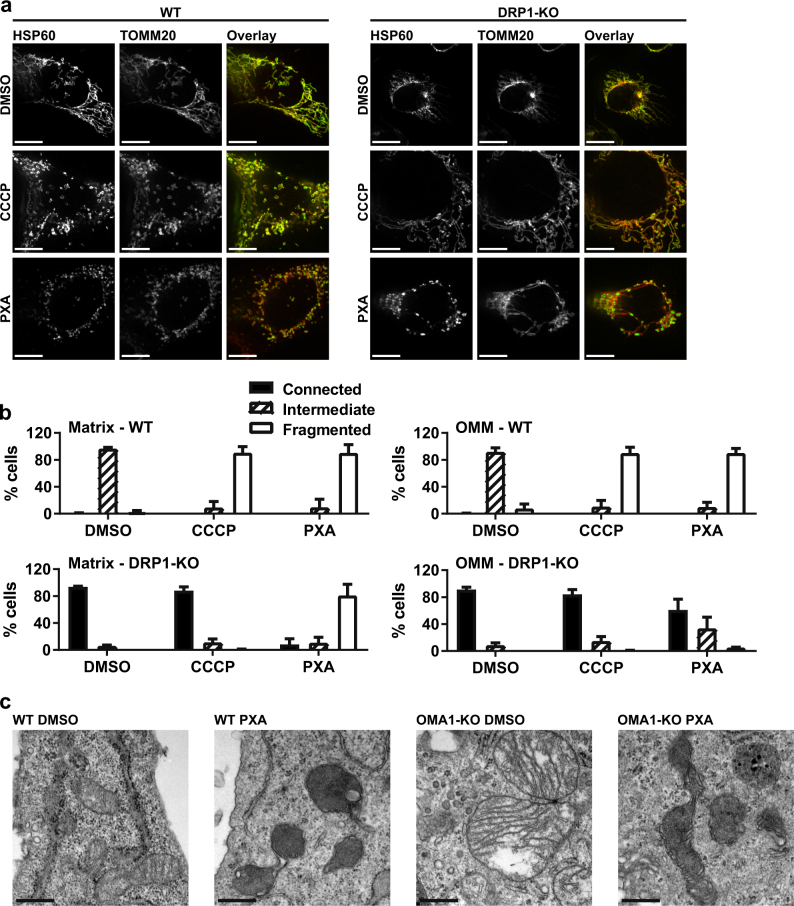
Effect of PXA on mitochondrial matrix morphology. **a** Confocal images of MEF cells (WT and DRP1-KO) at 30 min after treatment with either 0.1% v/v DMSO (vehicle control), 10 µM CCCP (positive control for fragmentation) or 10 µM PXA. HSP60 (green) was stained as a marker for the mitochondrial matrix, and TOMM20 (red) was stained as a marker for the outer mitochondrial membrane (OMM). Scale bars are equivalent to 10 µm. **b** Quantification of mitochondrial morphology by classification of 200 individual cells in each of three independent experiments; error bars = SD. **c** Transmission electron microscopy images of MEF cells (WT or OMA1-KO) after treatment with either 10 µM PXA or with 0.1% v/v DMSO (vehicle control) for 30 min. Scale bars are equivalent to 0.5 µm

Mitochondrial fission is also regulated by the dynamin DRP1, which mediates OMM fission. We, thus, tested the effect of PXA on the mitochondrial morphology in DRP1-deficient MEF cells. Again, fragmentation was observed within minutes after treatment (Fig. 7c, right panel; Supplementary Movie S8). We next used dual staining of both the matrix (via HSP60) as well as the OMM (via TOMM20) to determine whether both or only one of these structures are affected by PXA. CCCP was used as a positive control for fragmentation. As expected, CCCP could not induce fragmentation in DRP1-KO cells, and in WT cells it induced fragmentation of both the IMM and OMM together (Fig. 8a, b). In contrast, in the WT cells treated with PXA, fragmentation was much stronger and resulted in smaller fragments. More intriguingly, in DRP1-KO cells treated with PXA, only the matrix appeared to have fragmented, whereas the OMM appeared to have shrunken around the matrix fragments but otherwise remained connected (Fig. 8a, b). This effect could also be observed in cells deficient for both DRP1 and OPA1 (Fig. S6) demonstrating that PXA acts independently of canonical regulators of mitochondrial fission. Finally, a close examination of the mitochondrial ultrastructure by transmission electron microscopy (TEM) revealed that PXA causes OMA1-independent disruption of mitochondrial matrix morphology, complete loss of cristae, and condensation of IMM structures at the OMM (Fig. 8c).

Taken together, our results show that PXA disturbs mitochondrial form and function in several ways. Some effects, such as the rapid inhibition of both ΔΨ_m_ and the ETC at the same time, the delayed release of mitochondrial Ca^2+^, and the fragmentation of the inner but not the outer mitochondrial membrane, are unique and indicate a mode of action that is distinct from all other compounds to which it was compared in this study.

## Discussion

The mycotoxin PXA is a toxic natural product whose mechanism of action has so far remained elusive. We provide evidence that PXA disrupts mitochondrial function and causes IMM fragmentation and cristae disruption independently of DRP1 and OPA1, leading to the release of pro-apoptotic factors and ultimately to apoptosis.

PXA, just like CCCP, dissipates ΔΨ_m_ within seconds. In the case of CCCP, respiration increases to compensate this, whereas PXA has the opposite effect and blocks respiration. Conversely, respiration is also blocked by ETC inhibitors, yet in contrast to PXA, these do not strongly affect ΔΨ_m_. This suggests an entirely different mode of action for PXA. Additionally, unlike any of these compounds, PXA causes a strong mitochondrial release of Ca^2+^ and rapid fragmentation of the IMM but not the OMM.

Release of mitochondrial Ca^2+^ and loss of ΔΨ_m_ can be results of persistent mPTP opening, yet we showed that PXA does not strongly affect the mPTP. Since mitochondrial ion gradients are interdependent through various antiporters that are generally linked to ΔΨ_m_ (ref. ^2^), one might assume that loss of ΔΨ_m_ disturbs these gradients sufficiently to induce a net Ca^2+^ efflux from the mitochondria. For CCCP, contradictory results have been reported—in some cases it caused Ca^2+^ release^21^, in some cases it did not^22^. We observed no effect of CCCP and most ETC inhibitors on [Ca^2+^]_cyt_, and in fact both ETC-deficient (*ρ*^0^) mitochondria^23^ and depolarised mitochondria^24^ can still facilitate a net uptake of Ca^2+^. It, thus, appears that the mitochondrial Ca^2+^ release induced by PXA is not necessarily a result of its effects on the ETC and ΔΨ_m_, but rather that all of these effects might have a common cause.

The delay between addition of PXA and the first observable increase in [Ca^2+^]_cyt_ and decrease in [Ca^2+^]_mito_ contrasts with the immediate change in ΔΨ_m_ (Fig. 3d). This discrepancy could possibly be explained by the hypothesis that mitochondria release Ca^2+^ mainly into the cristae, and that the cristae junctions may function as bottlenecks for mitochondrial Ca^2+^ transport^8,25^. Cristae junctions are regulated by OPA1, which is cleaved by OMA1 in response to mitochondrial stress, leading to cristae disruption^26^. We not only showed that OPA1 is irreversibly cleaved by OMA1 upon PXA treatment, but that PXA also causes cristae disruption independently of OMA1. Thus, PXA-induced cristae disruption might cause the release of Ca^2+^ from the cristae and thus eventually into the cytosol. In addition, irreversible cristae disruption could well be a sufficient condition for the release of pro-apoptotic factors and thus the induction of apoptosis^26–28^.

OPA1 also affects the fragmentation of mitochondrial network structures, which can be induced by PXA as well as CCCP. In the case of CCCP, this is commonly explained by the excessive activation of DRP1 after increased OPA1 processing by OMA1, which in turn is a response to several kinds of cellular stress including impaired ATP production and loss of ΔΨ_m_ (ref. ^4,6,20,29–32^). Since PXA dissipates ΔΨ_m_, inhibits ATP production and consequently induces OMA1-mediated OPA1 cleavage, one might assume that it induces fragmentation via the same mechanism, yet we observed PXA-induced fragmentation events that were independent of DRP1, OMA1 and even OPA1.

Mitochondrial fragmentation independent of DRP1 is an unusual phenomenon but has been reported in cells undergoing apoptosis after pro-apoptotic factors had already been released^33,34^. In the case of PXA, however, fragmentation occurs within minutes after treatment, whereas pro-apoptotic factors are released only after several hours. In addition, whereas both the OMM and IMM are divided together during DRP1-dependent fragmentation, PXA can cause exclusive fragmentation of the IMM while the OMM remains intact. This is surprising since no active mechanism for exclusive IMM fission is known in higher eukaryotes, and there are only few reports documenting this phenomenon^6,35,36^.

The OMA1-processed short OPA1 forms play a role in IMM fission and cristae morphology^5,29,35^. However, since PXA-induced IMM fragmentation and cristae disruption are independent of both OMA1 and OPA1, this implies that OPA1 may well be an IMM fission regulator but not necessarily a fission executor. It has been recently proposed that OPA1 is dispensable for cristae junction biogenesis but may still be required for cristae junction remodelling^37^. Our results suggest that excessive OPA1 processing may be sufficient but not necessary for inner membrane remodelling and cristae disruption and for the consequent release of pro-apoptotic factors.

The independence of PXA-induced IMM fragmentation from DRP1, OMA1 and OPA1, as well as its very fast onset, suggest that it might not depend on the fission/fusion machinery at all, but could work via a completely separate mechanism. Since no such mechanism is known in higher eukaryotes, any attempt at explaining this effect remains speculative. One explanation could be a change in IMM fluidity or matrix architecture, causing an immediate and strong retraction of the IMM. This could result from interference with the tethering of IMM and OMM at mitochondrial contact sites. If this is the case, a possible mechanistic target of PXA could be the mitochondrial phospholipid cardiolipin, which is present almost exclusively in the IMM and especially at mitochondrial contact sites^38,39^. Cardiolipin serves as a membrane anchor for many proteins that are implicated in mitochondrial contact site formation, mitochondrial ultrastructure and the ETC, such as MIC27 (APOOL)^40,41^, F_1_F_O_ ATP synthase^41,42^, CYCS^41^, and ETC complexes III and IV^41,43,44^. A disruptive interaction between PXA and either cardiolipin or cardiolipin-binding proteins might thus explain several of the effects induced by PXA.

In summary, we identified PXA as a mitochondrial toxin with a mode of action distinct from known ETC inhibitors, OXPHOS uncouplers, and ionophores. Its effects, such as the rapid inhibition of both ETC and ΔΨ_m_, the release of mitochondrial Ca^2+^, and the induction of DRP1- and OPA1-independent cristae disruption and fission of the inner but not the outer mitochondrial membrane, might render it a useful tool in studying these phenomena. Further studies may reveal the molecular target of PXA and the mechanisms through which it induces mitochondrial Ca^2+^ release and IMM fission.

## Material and methods

### Cell lines and cell culture

Jurkat cells were obtained from DSMZ (#ACC-282). Ramos cells were kindly provided by Michael Engelke (Institute of Cellular and Molecular Immunology, University Hospital Göttingen, Göttingen, Germany). HeLa cells stably expressing mito-DsRed were kindly provided by Aviva M. Tolkovsky (Department of Clinical Neurosciences, University of Cambridge, England, UK) and have been described previously^45^. MEF cells deficient for OMA1 and/or YME1L1 as well as the corresponding wild-type cells were generated by Ruchika Anand and kindly provided by Thomas Langer (Institute for Genetics, University of Cologne, Germany) and have been described previously^29^. MEF cells deficient for DRP1 as well as the corresponding wild-type cells used for live imaging were kindly provided by Hiromi Sesaki (Department of Cell Biology, Johns Hopkins University, Baltimore, MD, USA) and have been described previously^33^. MEF cells deficient for DRP1 used for imaging of fixed cells were generated using the CRISPR/Cas9 system as described previously^46^. The DNA target sequence for the guide RNA was 5'-CAGTGGGAAGAGCTCAGTGC-3'. HCT116 cells were kindly provided by Frank Essmann (Interfaculty Institute of Biochemistry, Eberhard Karls University Tübingen, Germany). Transient expression of SMAC-mCherry and GFP-BAX was achieved by lipofection at 70–80% confluence using Lipofectamine 2000 (Life Technologies, Darmstadt, Germany). Cells were incubated with 0.15 µl Lipofectamine 2000, 50 ng pcDNA3-Smac(1-60)mCherry (Addgene ID 40880; this plasmid was kindly provided by Stephen Tait (Beatson Institute, University of Glasgow, Scotland, UK) and has been described previously^47^), and 50 ng pGFP-Bax (kindly provided by Nathan R. Brady, Department of Molecular Microbiology and Immunology, Johns Hopkins University, Baltimore, MD, USA) per well in glass bottom 8-well chambers (Ibidi, Planegg, Germany) for 16 h. HeLa cells used for Ca^2+^ measurements were cultured in Dulbecco’s modified Eagle’s medium supplemented with 10% fetal calf serum (FCS) and 4 mM l-glutamine, 100 U/ml penicillin and 100 μg/ml streptomycin at 37 °C and 5% CO_2_. They were authenticated using autosomal STR profiling performed by the University of Arizona Genetics Core and they fully matched the DNA fingerprint present in reference databases. Cell lines stably expressing either mito-DsRed (except HeLa; see above) or ratiometric mito-Pericam were generated by retroviral transfection using the Platinum-E (Plat-E) packaging cell line (kindly provided by Toshio Kitamura, Institute of Medical Science, University of Tokyo, Japan) and the retroviral vectors pMSCVpuro-mito-DsRed1 (Addgene ID 87379) or pMSCVpuro-mito-Pericam (Addgene ID 87381). The medium used for the cultivation of Jurkat cells and Ramos cells was RPMI 1640 medium, and the medium used for cultivation of HCT116 cells was McCoy’s 5A medium. All other cells were cultivated in high-glucose Dulbecco's Modified Eagle's medium (DMEM). All media were supplemented with 10% FCS, 100 U/ml penicillin, and 100 µg/ml streptomycin. All cell lines were maintained at 37 °C and 5% CO_2_ in a humidity-saturated atmosphere.

### Reagents

Phomoxanthone A was isolated and purified as described previously^16^. We found that PXA becomes unstable if dissolved in dimethyl sulfoxide (DMSO) and readily isomerises into the essentially non-toxic compound dicerandrol C (data not shown), in a process similar to the one previously described for the structurally related secalonic acids^48^. However, PXA is barely soluble in EtOH and not soluble in H_2_O. Therefore, PXA was prepared in small lyophilised aliquots and only dissolved in DMSO immediately before usage.

The tyrosine phosphatase inhibitor pervanadate (VO_4_^3−^) was freshly prepared by mixing 30 mM sodium orthovanadate with 60 mM H_2_O_2_ in phosphate-buffered saline (PBS) and incubating at room temperature (RT) in the dark for 10 min; sodium orthovanadate was purchased from Sigma (Munich, Germany), #450243; IM from Sigma, #I9657; thapsigargin (TG) from Sigma, #T9033; carbonyl cyanide *m*-chlorophenyl hydrazone (CCCP) from Sigma, #C2759; rotenone from Sigma, #45656; thenoyltrifluoroacetone (TTFA) from Sigma, #88300; antimycin A from Sigma, #A8674; sodium azide (NaN_3_) from Sigma, #S2002; oligomycin A from Toronto Research Chemicals (Toronto, Canada), #O532970; staurosporine (STS) from LC Laboratories (Woburn, MA, USA), #9300. All cell culture reagents were purchased from Life Technologies, and all other reagents where no manufacturer is explicitly mentioned were purchased from Carl Roth GmbH (Karlsruhe, Germany).

### Replicates and statistical analysis

Experiments were replicated at least three times, and representative data are shown. Error bars indicate standard deviation. All statistical analysis was performed using Prism v7.01 (GraphPad Software, La Jolla, CA, USA).

### In vitro kinase activity screening

The effect of PXA on the activity of 141 protein kinases was assessed by the International Centre for Kinase Profiling (Dundee, Scotland, UK) using a radioactive filter binding assay with ^33^P ATP^49,50^.

### Live measurement of [Ca^2+^]_cyt_ by Fluo-4-AM

Cells were stained by incubation in growth medium containing 1 µM Fluo-4-AM (Life Technologies; #F14201), 0.005% w/v Pluronic F-127 (Sigma, #540025), 10 mM HEPES and 5% v/v FCS at 30 °C. After 25 min, an equal volume of full growth medium was added, the temperature was increased to 37 °C, and the cells were incubated for another 10 min. After that, the cells were washed and resuspended in Krebs-Ringer buffer (10 mM HEPES pH 7.0, 140 mM NaCl, 4 mM KCl, 1 mM MgCl_2_, 10 mM glucose) supplemented with 1 mM CaCl_2_. The cells were kept at RT in the dark until measurement. Just before measurement, the cells were washed and resuspended in Krebs-Ringer buffer supplemented with 0.5 mM EGTA. Fluo-4-AM fluorescence was measured live using an LSRFortessa flow cytometer (BD, Franklin Lakes, NJ, USA) recording fluorescence in the FITC channel (Ex 488 nm, Em 530 ± 30 nm). For each sample, after at least 30 s of baseline measurement, the stimulus was added and measurement was continued for at least 10 min.

### Live measurement of [Ca^2+^]_mito_ and [Ca^2+^]_ER_ by CEPIA

Measurements of [Ca^2+^]_mito_ and [Ca^2+^]_ER_ in HeLa single cells were performed as described previously^51,52^, using the genetically-encoded Ca^2+^ indicators CEPIA3mt (Addgene ID 58219) and G-CEPIA1er (Addgene ID 58215), respectively, which were developed by Dr. M. Iino (The University of Tokyo, Japan)^53^. The constructs were introduced into HeLa cells utilising the X-tremeGENE HP DNA transfection reagent (Roche, Mannheim, Germany) according to the manufacturer’s protocol. The [Ca^2+^] measurements were performed 48 h after transfection using a Zeiss Axio Observer Z1 Inverted Microscope equipped with a 20 × air objective and a high-speed digital camera (Axiocam Hsm, Zeiss, Jena, Germany). Changes in fluorescence were monitored in the GFP channel (Ex 480 nm, Em 520 nm). Extracellular Ca^2+^ was chelated with 3 mM EGTA, and PXA (10 μM) or thapsigargin (1 μM) were added as indicated on the figures. All traces were normalised (*F*/*F*_0_) where *F*_0_ is the starting fluorescence of each trace.

### Live measurement of [Ca^2+^]_mito_ by ratiometric mito-Pericam

Ramos cells stably transfected with ratiometric mito-Pericam as described above were used for this measurement. Ratiometric mito-Pericam is a Ca^2+^-sensitive fluorescent protein and was described previously^54,55^. An increase in [Ca^2+^] causes a shift of the Pericam excitation maximum from ~410 to ~495 nm while the emission peak remains at ~515 nm. Pericam fluorescence was measured live using an LSRFortessa flow cytometer recording fluorescence in both the FITC channel (Ex 488 nm, Em 530 ± 30 nm) and the AmCyan channel (Ex 405 nm, Em 525 ± 50 nm). For each sample, after at least 30 s of baseline measurement, the stimulus was added and measurement was continued for at least 10 min. The ratio of fluorescence with excitation at 488 to 405 nm was calculated.

### Live measurement of mPTP opening by cobalt-calcein assay

This method was adapted from previously published protocols^10,18,56^. The cells were stained by incubation in Krebs-Ringer buffer supplemented with 1 mM CaCl_2_, 1 mM CoCl_2_, and 1 µM calcein-AM (Life Technologies, #65-0853-78) at 37 °C for 30 min. After that, the cells were washed and maintained in Krebs-Ringer buffer supplemented with 1 mM CaCl_2_ and 1.6 µM cyclosporin H (CsH) to prevent passive efflux of calcein. For live measurement by confocal microscopy, imaging and quantification were performed using a Perkin Elmer Spinning Disc microscope with a 60× objective (oil-immersion and NA = 1.49) at an excitation wavelength of 488 nm. The videos were obtained at 1000 × 1000 pixel resolution with a Hamamatsu C9100 camera. Additional live measurement by flow cytometry was performed using an LSRFortessa flow cytometer recording fluorescence in the FITC channel (Ex 488 nm, Em 530 ± 30 nm). For each sample, after at least 30 s of baseline measurement, the stimulus was added and measurement was continued for at least 10 min.

### Isolation of live mitochondria

Adherent cells were harvested by a cell scraper. All cells were pelletised by centrifugation at 600 rcf, resuspended in ice-cold mitochondria isolation buffer (210 mM mannitol, 70 mM sucrose, 1 mM K_2_EDTA, 20 mM HEPES), and passed through a 23 G needle ten times. The resulting suspension was centrifuged at 600 rcf and the supernatant was transferred to a new tube and centrifuged at 6500 rcf and 4 °C for 15 min. The resulting mitochondrial pellet was resuspended in sodium-free mitochondrial respiration buffer MiR05 (0.5 mM EGTA, 3 mM MgCl_2_, 60 mM lactobionic acid, 20 mM taurine, 10 mM KH_2_PO_4_, 20 mM HEPES, 110 mM d-sucrose, 0.1% w/v fatty-acid-free bovine serum albumin [BSA]) supplemented with 10 mM succinate and 5 mM malate.

### Live measurement of mitochondrial Ca^2+^ retention capacity by calcium green

Live mitochondria isolated as described above were stained by incubation in MiR05 buffer supplemented with 10 mM succinate, 5 mM malate, and 1 µM calcium green AM (Life Technologies, #C3012) for 20 min on a shaker at 37 °C. Before measurement, the mitochondria were pelletised at 6500 rcf for 5 min, washed and resuspended in MiR05 supplemented with 10 mM succinate, 5 mM malate, and 5 µM of either CsH or CsA. In experiments where the mitochondria were loaded with Ca^2+^ before measurement, this was achieved by incubation in MiR05 additionally supplemented with 150 µM CaCl_2_ on a shaker at 37 °C for 10 min after the first washing step and followed by a second washing step.

### Measurement of mitochondrial membrane potential by TMRE and TMRM

For measurement in whole cells, the cells were stained by incubation in full growth medium containing 100 nM tetramethylrhodamine ethyl ester (TMRE; AAT Bioquest, Sunnyvale, CA, USA; #22220) and 10 mM HEPES at 37 °C in the dark for 15 min. After that, the cells were washed and resuspended in full growth medium containing 10 mM HEPES and were incubated at 37 °C in the dark for another 15 min. The cells were maintained at these conditions until measurement. For measurement in live mitochondria, these were isolated as described above, resuspended in sodium-free mitochondrial respiration buffer MiR05 supplemented with 10 mM succinate, 5 mM malat, and 1 mM ADP, stained with 50 nM tetramethylrhodamine methyl ester (TMRM; Life Technologies, #T668) at 37 °C for 15 min, and washed and resuspended in MiR05 additionally supplemented with 1.6 µM cyclosporin H (CsH) to prevent passive TMRM leakage. For live measurement, TMRE or TMRM fluorescence was measured using an LSRFortessa flow cytometer recording fluorescence in the PE channel (Ex 488 nm, Em 575 ± 26 nm). For each sample, after at least 30 s of baseline measurement, the stimulus was added and measurement was continued for at least 10 min. For the titration of the EC_50_ for mitochondrial depolarisation, TMRE fluorescence was measured using a Synergy Mx microplate reader (BioTek, Bad Friedrichshall, Germany) recording fluorescence at Ex 549 ± 9 nm, Em 575 ± 9 nm. TMRE fluorescence was measured right before and 10 min after addition of PXA. EC_50_ values were calculated using Prism v7.01.

### Live O_2_ respirometry measurements

This method was adapted from previously published protocols^19,57^. All measurements were performed using an OROBOROS Oxygraph-2k (Oroboros Instruments, Innsbruck, Austria). For measurement of total cellular respiration, intact cells (2 × 10^6^ cells/ml) were used and maintained in full growth medium supplemented with 20 mM HEPES during measurement. For direct measurement of mitochondrial respiration, digitonin-permeabilised cells (2 × 10^6^ cells/ml) were used and maintained in mitochondrial respiration buffer MiR05 during measurement. To induce respiration, 10 mM glutamate, 5 mM malate, 1 mM ADP, and 5 µg/ml digitonin were added. The following complex-specific ETC inducers were used: For complex II, 10 mM succinate (from Sigma, #S3674); for complex III, 1 mM tetramethylhydroquinone / duroquinol (from TCI Germany, Eschborn, Germany; #T0822); for complex IV, 50 µM tetramethyl-*p*-phenylenediamine (TMPD; from Sigma, #87890) supplemented with 200 µM ascorbate.

### Fluorimetric O_2_ consumption assay

This measurement was performed using the *MITO-ID® Extracellular O2 Sensor Kit (High Sensitivity)* (Enzo Life Sciences, Lörrach, Germany; #51045) according to manufacturer’s instructions. Fluorescence was measured using a Synergy Mx microplate reader (Ex 340–400 nm, Em 630–680 nm; time-resolved fluorescence, delay time 30 µs, integration time 100 µs).

### Measurement of cellular ATP levels

This measurement was performed using the *Mitochondrial ToxGlo™ Assay* (Promega, Mannheim, Germany; #G8000) according to manufacturer’s instructions. Since most cancer cells prefer ATP synthesis by glycolysis over OXPHOS if glucose is present, this experiment was conducted in the presence of either glucose or galactose as the only available sugar, the latter of which reduces the net ATP yield of glycolysis to zero and forces the cells to resort to OXPHOS for ATP production^58,59^.

### Fluorimetric caspase-3 activity assay

Caspase-3 activity was measured as described previously^60^. Briefly, cells were harvested by centrifugation at 600 rcf and lysed with 50 μl of ice-cold lysis buffer (20 mM HEPES, 84 mM KCl, 10 mM, MgCl_2_, 200 μM EDTA, 200 μM EGTA, 0.5% NP-40, 1 µg/ml leupeptin, 1 μg/ml pepstatin, 5 μg/ml aprotinin) on ice for 10 min. Cell lysates were transferred to a black flat-bottom microplate and mixed with 150 μl of ice-cold reaction buffer (50 mM HEPES, 100 mM NaCl, 10% sucrose, 0.1% CHAPS, 2 mM CaCl_2_, 13.35 mM DTT, 70 μM Ac-DEVD-AMC). The kinetics of AMC release were monitored by measuring AMC fluorescence intensity (Ex 360 nm, Em 450 nm) at 37 °C in intervals of 2 min over a time course of 150 min, using a Synergy Mx microplate reader. The slope of the linear range of the fluorescence curves (Δrfu/min) was considered as corresponding to caspase-3 activity.

### Measurement of cell viability by MTT assay

Cell viability was determined by the ability to convert the yellow MTT substrate (Roth, #4022) into a blue formazan product. MTT solution (5 mg/ml MTT in PBS) was added to cells to a final concentration of 1 mg/ml, and the cells were then incubated at 37 °C for 60 min and pelletised at 600 rcf. The supernatant was discarded and replaced with DMSO. After the formazan crystals were fully dissolved, absorption was measured (test wavelength 570 nm, reference wavelength 650 nm). Reference absorbance was subtracted from test absorbance. Cell-free medium samples were considered as having 0% viability and the average of the control samples was considered as having 100% viability. IC_50_ values were calculated using Prism v7.01.

### Immunoblotting

Cells were harvested by centrifugation at 11,000 rcf in 4 °C for 10 s, quick-frozen in liquid nitrogen, thawed on ice, incubated in lysis buffer (20 mM Tris-HCl, 150 mM NaCl, 1% v/v Triton X-100, 0.5 mM EDTA, 1 mM Na_3_VO_4_, 10 mM NaF, 2.5 mM Na_4_P_2_O_7_, 0.5% sodium deoxycholate, protease inhibitor (Sigma, #P2714)) for 30 min and vortexed repeatedly. The cell lysates were then cleared from cell debris by centrifugation at 20,000 rcf for 15 min. Sodium dodecyl sulfate-polyacrylamide gel electrophoresis and western blot were performed according to standard protocol. The antibodies used for protein detection were mouse anti-phospho-tyrosine (Merck-Millipore, Darmstadt, Germany; clone 4G10, #05-1050); rabbit anti-OPA1 (described previously^37^); mouse anti-ACTB (Sigma; clone AC-74, #A5316); and mouse anti-VCL (Sigma; clone hVIN-1, #V9131).

### Confocal microscopy

Live imaging of HCT116 cells transiently expressing GFP-BAX and SMAC-mCherry was performed using a Zeiss LSM 710 ConfoCor3 microscope (Carl Zeiss, Jena, Germany) with a C‐Apochromat × 40 N.A. 1.2 water immersion objective (Zeiss). Excitation light came from argon ion (488 nm) and DPSS (561 nm) lasers. The cells were maintained in full growth medium at 37 °C and 5% CO_2_ during imaging. Images were recorded every 5 min and were processed with Fiji^61^. For each time frame, the standard deviation (SD) of the fluorescence intensity was measured for each channel. A low SD was considered as corresponding to homogenous distribution, whereas a high SD was considered as corresponding to accumulation.

Live imaging of HeLa cells stably expressing mito-DsRed was performed using a Cell Observer SD Dual Cam spinning disc confocal microscope (Zeiss) equipped with a C-Apochromat 63×, N.A. of 1.45 oil-immersion objective. Excitation light came from an argon ion (488 nm) and DPSS (561 nm) laser. The cells were maintained in full growth medium supplemented with 10 mM HEPES at 37 °C during imaging. Images were recorded every 5 s.

Live imaging of MEF cells stably expressing mito-DsRed was performed using a Perkin Elmer Spinning Disc microscope with a 60 × objective (oil-immersion and NA = 1.49) at an excitation wavelength of 561 nm. The videos were obtained at 1000 × 1000 pixel resolution with a Hamamatsu C9100 camera. The cells were maintained in full growth medium supplemented with 10 mM HEPES at 37 °C during imaging.

For imaging of fixed HeLa and MEF cells, the cells were seeded on glass coverslips and grown to 60–90% confluence prior to experiments. Cells were treated with either 10 μM PXA, 10 μM CCCP or 0.1% v/v DMSO for 30 min, and were fixed by incubation with pre-warmed 4% paraformaldehyde in PBS at 37 °C for 10 min. Coverslips were then washed once with PBS, followed by incubation with PBS supplemented with 0.5% Triton X-100 for 10 min at RT. The coverslips were washed three times for 3–5 min with PBS supplemented with 0.2% Tween-20 (PBS-T). The coverslips were then incubated at RT for 30 min with blocking buffer (PBS-T supplemented with 0.2% fish gelatin and 5% goat serum) in a humidified box, followed by 1 h incubation with primary antibodies (anti-HSP60 clone N-20, #sc-1052 and anti-TOMM20 clone FL-145, #sc-11415 both from Santa Cruz, Dallas, TX, USA) diluted in blocking buffer. The coverslips were then washed three times with PBS-T, and incubated with blocking buffer for 30 min before adding secondary antibodies (Alexa Fluor 488-labelled donkey anti-goat and Alexa Fluor 594-labelled donkey anti-rabbit). Immunofluorescence images were acquired with a Marianas spinning disc confocal microscope (Intelligent Imaging Innovations, Denver, CO, USA).

### Transmission electron microscopy

TEM samples were fixed for a minimum of 4 h in 2.5% v/v glutaraldehyde (GA) and 4% w/v paraformaldehyde (PFA) in 0.1 M cacodylate buffer (pH 7.4) at 4 °C. Then, samples were incubated in 1% osmium tetroxide in 0.1 M cacodylate buffer for 2 h. Dehydration was achieved using acetone (50%, 70%, 90% and 100%) and block contrast was applied (1% phosphotungstic acid/0.5% uranylacetate in 70% acetone). A SPURR embedding kit (Serva, Heidelberg, Germany) was used to embed samples, which were polymerised overnight at 70 °C, before cutting into 80 nm sections using an Ultracut EM UC7 (Leica, Wetzlar, Germany). Images were captured using an H600 TEM (Hitachi, Tokyo, Japan) at 75 kV.

## Electronic supplementary material

Supplemental Table S1

Supplemental Figures S1, S2, S3

Supplemental Figures S4, S5

Supplemental Figure S6

Supplemental Movie S1

Supplemental Movie S2

Supplemental Movie S3

Supplemental Movie S4

Supplemental Movie S5

Supplemental Movie S6

Supplemental Movie S7

Supplemental Movie S8

Supplementary Information
