# Supplementary material for: The mycotoxin phomoxanthone A disturbs the form and function of the inner mitochondrial membrane
Source: Cell Death Dis. 2018 Feb 19;9(3):286. doi: 10.1038/s41419-018-0312-8 (PMC5833434; doi:10.1038/s41419-018-0312-8)
Supplement: Supplementary file 2 — Supplemental Figures S1, S2, S3 [file 41419_2018_312_MOESM2_ESM.pdf]

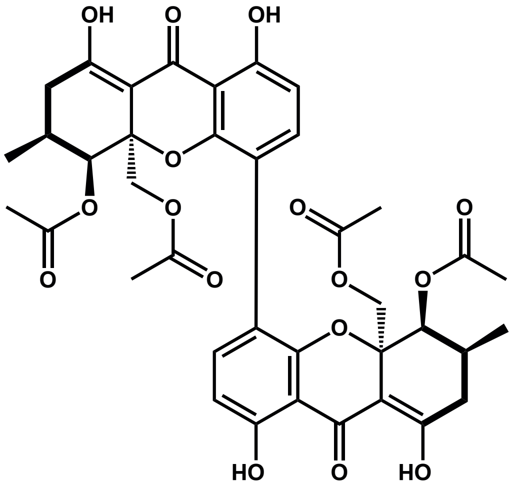

Figure S1 Structure of phomoxanthone A (PXA), adapted from Rönsberg et al. 2013 (PMID 24295452).

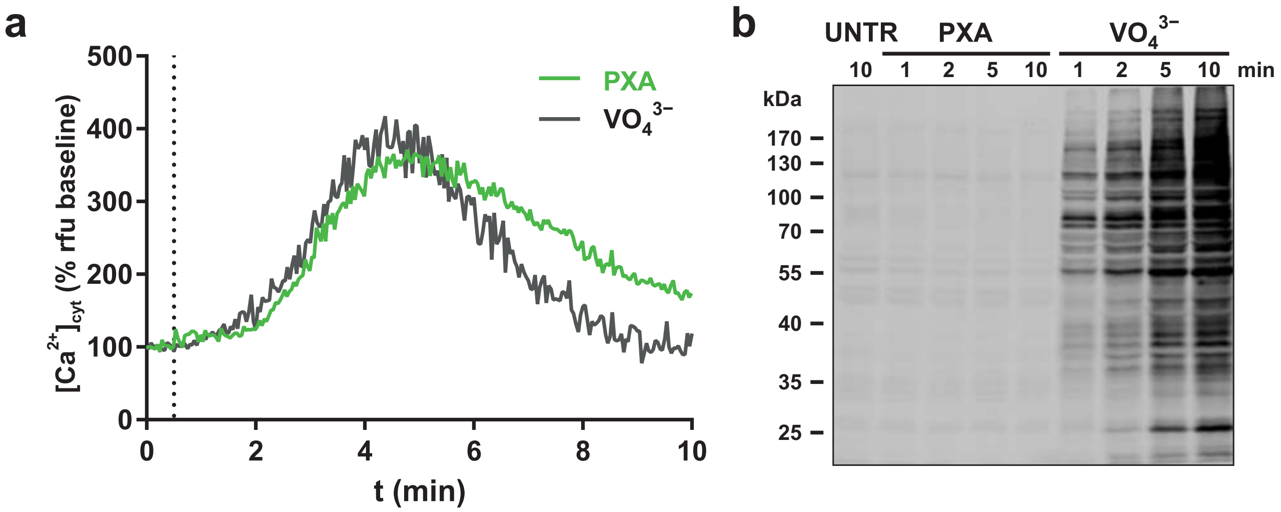

Figure S2 Comparison of PXA to the tyrosine phosphatase inhibitor pervanadate (VO_4^{3-}). (**a**) Induction of Ca^{2+} release from intracellular stores in Ramos cells after treatment with either PXA (10 µM) or VO_4^{3-} (30 µM). PXA Ca^{2+} trace adapted from Figure 1. (**b**) Effect of PXA (10 µM) and VO_4^{3-} (30 µM) on tyrosine phosphorylation in Ramos cells as detected by immunoblotting. Untreated cells (UNTR) were used as control.

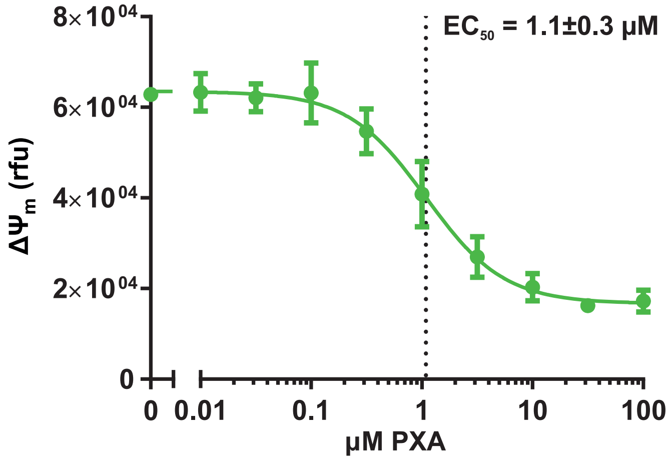

Figure S3 Titration of PXA to determine the EC_{50} for mitochondrial depolarisation (indicated by the dotted line), using the $\Delta\Psi_m$-sensitive fluorescent probe TMRE. Measurement was performed by a microplate reader after 10 min of treatment. Data are the means of three independent experiments; error bars = SD.
