# Supplementary material for: The mycotoxin phomoxanthone A disturbs the form and function of the inner mitochondrial membrane
Source: Cell Death Dis. 2018 Feb 19;9(3):286. doi: 10.1038/s41419-018-0312-8 (PMC5833434; doi:10.1038/s41419-018-0312-8)
Supplement: Supplementary file 3 — Supplemental Figures S4, S5 [file 41419_2018_312_MOESM3_ESM.pdf]

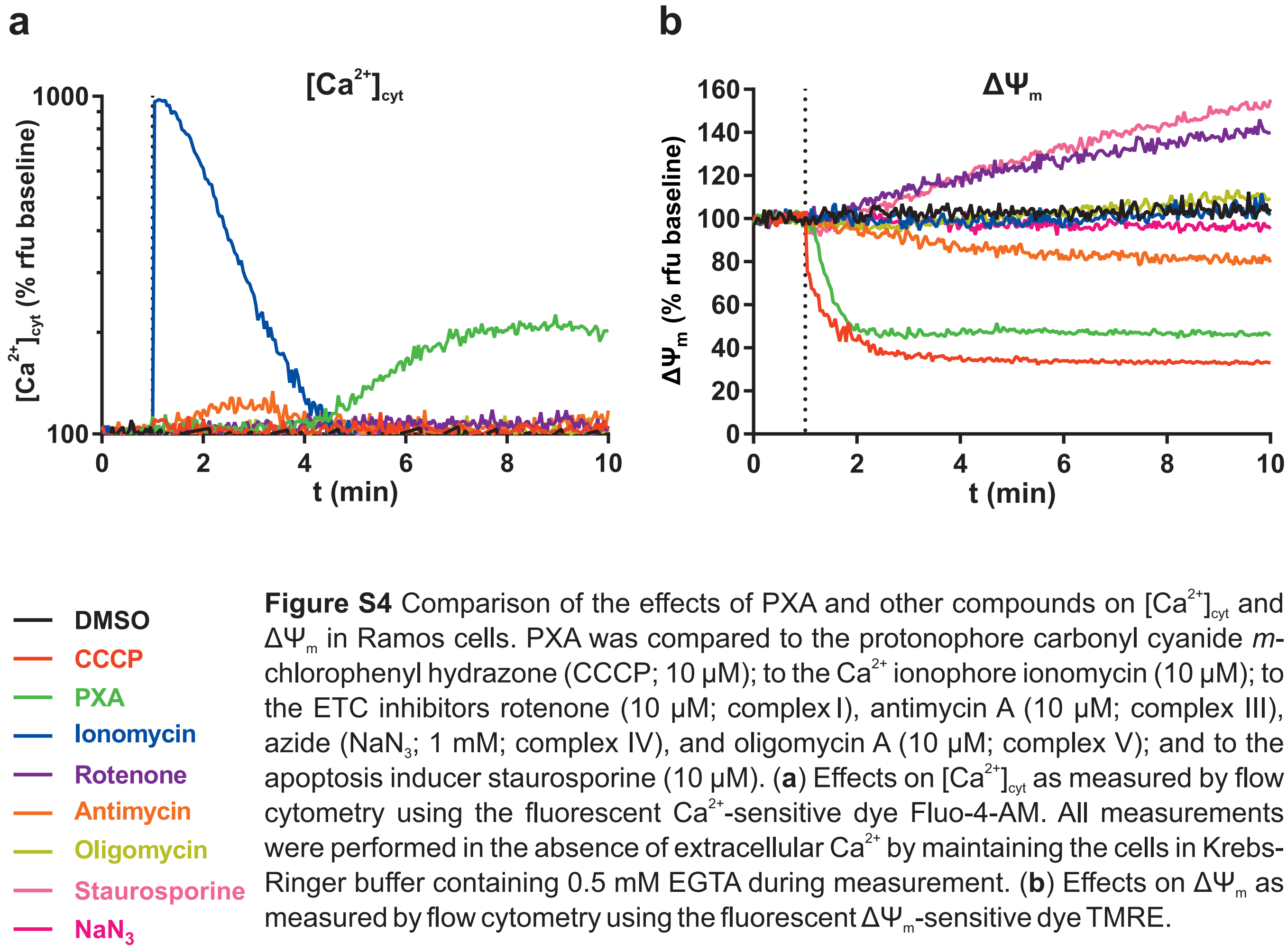

Figure S4 Comparison of the effects of PXA and other compounds on $[Ca^{2+}]_{cyt}$ and $\Delta\Psi_m$ in Ramos cells. PXA was compared to the protonophore carbonyl cyanide *m*-chlorophenyl hydrazone (CCCP; 10 µM); to the Ca^{2+} ionophore ionomycin (10 µM); to the ETC inhibitors rotenone (10 µM; complex I), antimycin A (10 µM; complex III), azide (NaN_3; 1 mM; complex IV), and oligomycin A (10 µM; complex V); and to the apoptosis inducer staurosporine (10 µM). (**a**) Effects on $[Ca^{2+}]_{cyt}$ as measured by flow cytometry using the fluorescent Ca^{2+}-sensitive dye Fluo-4-AM. All measurements were performed in the absence of extracellular Ca^{2+} by maintaining the cells in Krebs-Ringer buffer containing 0.5 mM EGTA during measurement. (**b**) Effects on $\Delta\Psi_m$ as measured by flow cytometry using the fluorescent $\Delta\Psi_m$-sensitive dye TMRE.

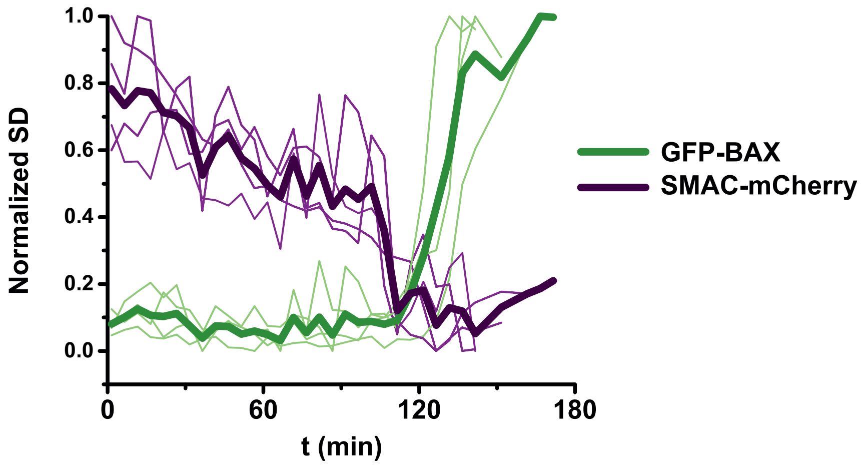

Figure S5 Quantification of the effects of PXA on mitochondrial BAX accumulation and SMAC release in HCT116 cells. The normalised standard deviation (SD) of the fluorescence intensity of GFP-BAX and SMAC-mCherry upon treatment with PXA (10 µM) in individual cells (N=4) was used as a measure of distribution inside the cells. A low SD corresponds to homogenous distribution while a high SD corresponds to accumulation. Thinner lines represent measurements of individual cells, while thicker lines represent the average of all recorded cells.
