# Supplementary material for: The mycotoxin phomoxanthone A disturbs the form and function of the inner mitochondrial membrane
Source: Cell Death Dis. 2018 Feb 19;9(3):286. doi: 10.1038/s41419-018-0312-8 (PMC5833434; doi:10.1038/s41419-018-0312-8)
Supplement: Supplementary file 4 — Supplemental Figure S6 [file 41419_2018_312_MOESM4_ESM.pdf]

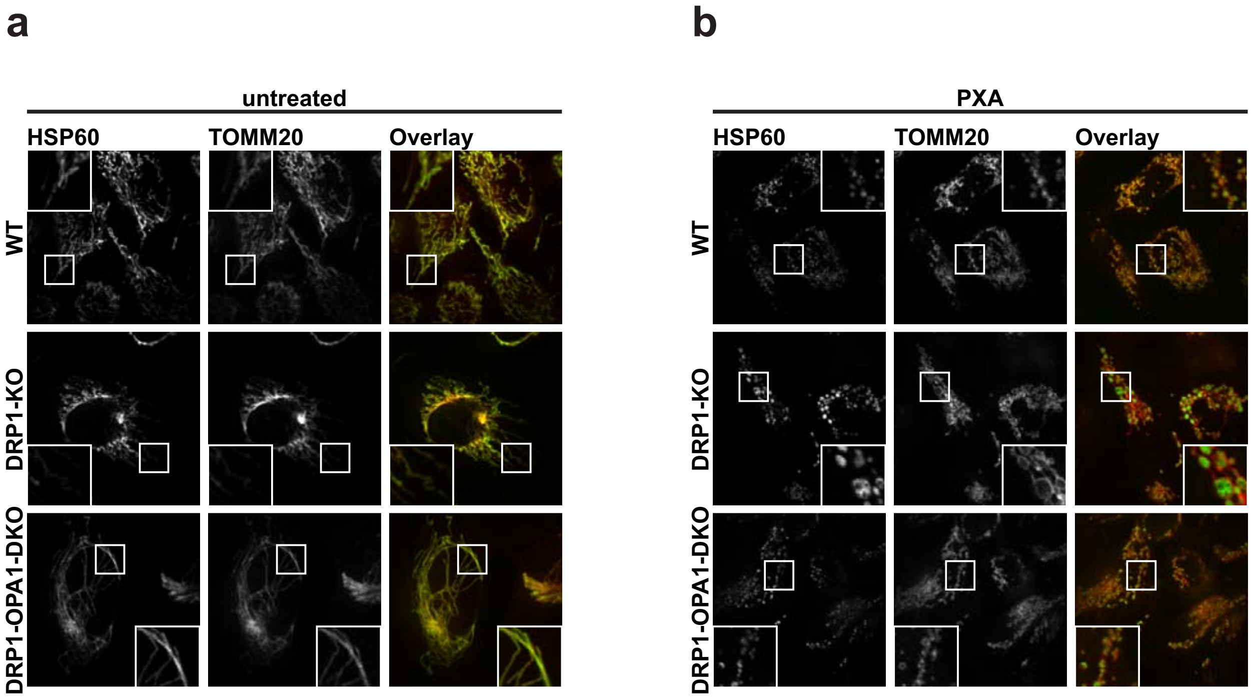

Figure S6 Confocal images of HeLa cells (WT, DRP1-KO, and DRP1-OPA1-DKO) at 30 min after treatment with either (**a**) 0.1% v/v DMSO (vehicle control) or (**b**) 10 μM PXA. HSP60 (green) was stained as a marker for the mitochondrial matrix, and TOMM20 (red) was stained as a marker for the outer mitochondrial membrane (OMM).
