# Supplementary material for: The mycotoxin phomoxanthone A disturbs the form and function of the inner mitochondrial membrane
Source: Cell Death Dis. 2018 Feb 19;9(3):286. doi: 10.1038/s41419-018-0312-8 (PMC5833434; doi:10.1038/s41419-018-0312-8)
Supplement: Supplementary file 13 — Supplementary Information [file 41419_2018_312_MOESM13_ESM.docx]

**Supplementary Information**

**Figure S1** Structure of phomoxanthone A (PXA), adapted from Rönsberg et al. 2013 (PMID 24295452).

**Figure S2** Comparison of PXA to the tyrosine phosphatase inhibitor pervanadate (VO_4_^3−^). (**a**) Induction of Ca^2+^ release from intracellular stores in Ramos cells after treatment with either PXA (10 µM) or VO_4_^3−^ (30 µM). PXA Ca^2+^ trace adapted from Figure 1. (**b**) Effect of PXA (10 µM) and VO_4_^3−^ (30 µM) on tyrosine phosphorylation in Ramos cells as detected by immunoblotting. Untreated cells (UNTR) were used as control.

**Figure S3** Titration of PXA to determine the EC_50_ for mitochondrial depolarisation (indicated by the dotted line), using the ΔΨ_m_-sensitive fluorescent probe TMRE. Measurement was performed by a microplate reader after 10 min of treatment. Data shown are the means of three independent experiments; error bars = SD.

**Figure S4** Comparison of the effects of PXA and other compounds on [Ca^2+^]_cyt_, ΔΨ_m_, and mPTP opening in Ramos cells. PXA was compared to the protonophore carbonyl cyanide *m*-chlorophenyl hydrazone (CCCP; 10 µM); to the Ca^2+^ ionophore ionomycin (10 µM); to the ETC inhibitors rotenone (10 µM; complex I), antimycin A (10 µM; complex III), azide (NaN_3_; 1 mM; complex IV), and oligomycin A (10 µM; complex V); and to the apoptosis inducer staurosporine (10 µM). (**a**) Effects on [Ca^2+^]_cyt_ as measured by flow cytometry using the fluorescent Ca^2+^-sensitive dye Fluo-4-AM. All measurements were performed in the absence of extracellular Ca^2+^ by maintaining the cells in Krebs-Ringer buffer containing 0.5 mM EGTA during measurement. (**b**) Effects on ΔΨ_m_ as measured by flow cytometry using the fluorescent ΔΨ_m_-sensitive dye TMRE.

**Figure S5** Quantification of the effects of PXA on mitochondrial BAX accumulation and SMAC release in HCT116 cells. The normalised standard deviation (SD) of the fluorescence intensity of GFP-BAX and SMAC-mCherry upon treatment with PXA (10 µM) in individual cells (N=4) was used as a measure of distribution inside the cells. A low SD corresponds to homogenous distribution while a high SD corresponds to accumulation. Thinner lines represent measurements of individual cells, while thicker lines represent the average of all recorded cells.

**Figure S6** Confocal images of HeLa cells (WT, DRP1-KO, and DRP1-OPA1-DKO) at 30 min after treatment with either (**a**) 0.1% v/v DMSO (vehicle control) or (**b**) 10 µM PXA. HSP60 (green) was stained as a marker for the mitochondrial matrix, and TOMM20 (red) was stained as a marker for the outer mitochondrial membrane (OMM).

**Table S1** Effect of PXA on the activity of various protein kinases in vitro.

**Movie S1** Confocal live imaging of a HeLa cell stained with 1 µM calcein AM in the presence of 1 mM CaCl_2_ and 1 mM CoCl_2_ and washed afterwards. DMSO (0.1% v/v) was added at t = 0.

**Movie S2** Confocal live imaging of a HeLa cell stained with 1 µM calcein AM in the presence of 1 mM CaCl_2_ and 1 mM CoCl_2_ and washed afterwards. PXA (10 µM) was added at t = 0.

**Movie S3** Confocal live imaging of a HeLa cell stained with 1 µM calcein AM in the presence of 1 mM CaCl_2_ and 1 mM CoCl_2_ and washed afterwards. IM (2 µM) was added at t = 0.

**Movie S4** Confocal live imaging of a HCT116 cell expressing GFP-BAX (green) and SMAC-mCherry (magenta). Scale bar equivalent to 10 µm. PXA was added at about t = 0.

**Movie S5** Confocal live imaging of a MEF cell (WT) stably expressing the fluorescent dye mito-DsRed, which localises to the mitochondrial matrix. DMSO (0.1% v/v) was added at about t = 0.

**Movie S6** Confocal live imaging of a MEF cell (WT) stably expressing the fluorescent dye mito-DsRed, which localises to the mitochondrial matrix. PXA (10 µM) was added at about t = 0.

**Movie S7** Confocal live imaging of a MEF cell (OMA1-YME1L1-DKO) stably expressing the fluorescent dye mito-DsRed, which localises to the mitochondrial matrix. PXA (10 µM) was added at about t = 20 s.

**Movie S8** Confocal live imaging of a MEF cell (DRP1-KO) stably expressing the fluorescent dye mito-DsRed, which localises to the mitochondrial matrix. PXA (10 µM) was added at about t = 9 min.
